# Experience in the Management of Genitourinary Syndrome of Menopause With Fractional CO2 Laser at Dr. Alejandro Dávila Bolaños Military Teaching Hospital in Nicaragua

**DOI:** 10.7759/cureus.74667

**Published:** 2024-11-28

**Authors:** Violeta López, María Esther Suárez Garcia, Karen S Rivera, Andres Rivera, Christopher Romero

**Affiliations:** 1 Obstetrics and Gynecology, Hospital Militar Escuela "Dr. Alejandro Dávila Bolaños", Managua, NIC; 2 School of Medicine, Hospital Militar Escuela "Dr. Alejandro Dávila Bolaños", Managua, NIC

**Keywords:** co2 fractional laser, dyspareunia, genitourinary syndrome of menopause, pruritus, quality-of-life

## Abstract

Objective: We evaluated the quality of life improvements in patients with genitourinary syndrome of menopause (GSM) who received fractional CO_2_ laser therapy at the Gynecology-Obstetrics Department of "Dr. Alejandro Dávila Bolaños" Military School Hospital (HMADB) in Managua, Nicaragua, from June 2022 to October 2023.

Materials and methods: This descriptive cross-sectional study included 25 GSM patients with contraindications or insufficient response to local estrogen therapy. Each patient received two sessions of fractional CO_2_ laser therapy targeting the vaginal canal, vestibule, and labia minora, with a six-week interval between treatments. The Vulvovaginal Symptom Questionnaire (VSQ) was administered pre-treatment and at three and six months post-treatment. Statistical analysis was performed using the paired t-test.

Results: Most patients were between 56 and 68 years of age (80%), and 56% were in active sexual relationships. Prior to CO_2_ laser therapy, 100% of participants reported vulvar symptoms, 92% experienced impacts on daily life, and 72% had difficulties in sexual life. Six months post-treatment, these percentages dropped to 40%, 16%, and 8%, respectively. Overall, 52% of patients became asymptomatic, while 48% reported mild symptoms. Paired t-test analysis indicated statistically significant improvements (p < 0.05) between pre- and post-treatment scores. Mild and transient adverse effects included pruritus (40%), pain (28%), dyspareunia (24%), and erythema (16%).

Conclusion: Fractional CO_2_ laser therapy is a safe and effective non-hormonal option for GSM management, with minimal and transient side effects, providing a valuable alternative for patients who cannot use estrogen-based treatments.

## Introduction

Genitourinary syndrome of menopause (GSM) is a chronic and progressive condition that affects the vulvovaginal area, sexual function, and the lower urinary tract in approximately 50% of women. It encompasses the spectrum of changes resulting from estrogen deprivation during menopause. Unlike vasomotor symptoms, which often improve over time, genitourinary symptoms tend to progress if left untreated and rarely resolve spontaneously. This progression can significantly impact a woman's quality of life, particularly regarding self-esteem and intimacy in relationships [1-3].

Diem and Danan further highlight that the most affected quality of life domain in women with GSM is sexual function, but other areas such as self-confidence, self-esteem, sleep, and general enjoyment of life are also impacted [4]. This underscores the broad and multifaceted nature of GSM's impact on quality of life. Selvi et al. found that Turkish postmenopausal women with GSM experienced worse quality of life scores in psychosocial and sexual domains, as measured by the Menopause-Specific Quality of Life Questionnaire and the King's Health Questionnaire [5].

The management of GSM includes both non-hormonal and hormonal therapies. Non-hormonal options such as vaginal lubricants and moisturizers are typically recommended for women with mild symptoms. For moderate to severe symptoms, low-dose vaginal estrogens, vaginal dehydroepiandrosterone (DHEA), systemic hormone therapy, and the estrogen agonist/antagonist ospemifene are effective treatments that are generally considered the "gold standard" [6]. Despite its efficacy, responses to hormone therapy may be inadequate, and it may be contraindicated in some patients or associated with safety concerns. This situation underscores the importance of exploring alternative therapeutic approaches, such as laser treatment [2].

Studies indicate significant improvements in GSM symptoms such as dryness, dyspareunia, itching, burning, and dysuria after carbon dioxide (CO_2_) laser therapy, with no major adverse events noted. Patient satisfaction was high, although no notable differences were observed in the Female Sexual Function Index (FSFI) and Vaginal Health Index (VHI) between CO_2_ laser therapy and sham groups [7]. A multicenter cohort study showed that both fractional CO_2_ laser and topical estrogen therapy significantly alleviated GSM symptoms, with benefits lasting 6-12 months post-treatment and no major adverse effects in either group [8].

Overall, CO_2_ laser therapy appears to offer a promising non-hormonal treatment option for GSM, with significant improvements in both symptoms and quality of life measures. However, the quality of evidence is still considered "low" or "very low" due to the heterogeneity of studies and the lack of long-term safety data. Nevertheless, no established protocols or clinical guidelines currently govern its use in this context. The objective of this study was to evaluate the improvement in quality of life among patients diagnosed with GSM who received fractional CO_2_ laser therapy. This evaluation was conducted using the Vulvovaginal Symptom Questionnaire (VSQ) to systematically assess changes in symptoms and overall well-being.

## Materials and methods

Study design

This was a descriptive cross-sectional study designed to evaluate the effectiveness of fractional CO_2_ laser treatment in patients with GSM.

Type, area, and period of study

The study was conducted in the Department of Gynecology and Obstetrics at Dr. Alejandro Dávila Bolaños Military Hospital (HMADB), Managua, Nicaragua, from June 2022 to October 2023.

Sample

The study included all patients with GSM treated with fractional vaginal CO_2_ laser in at least two sessions. The study included 25 patients who met specific criteria: diagnosis of GSM, prior unsatisfactory treatment with estrogen or contraindication due to a history of hormone-dependent cancer, completion of two sessions of fractionated CO_2_ laser therapy with a six-week interval targeting the vaginal canal, vestibule, and labia minora, and voluntary consent to participate. Patients without a GSM diagnosis, those on hormonal treatment at the study's start, those who did not complete both laser sessions, or those who declined to participate were excluded.

Therapies

Administered by trained personnel from the urogynecology service of the HMADB, the patients underwent two sessions with fractionated CO_2_ laser in the vagina, vaginal vestibule, and labia minora, with an interval of six weeks. The KLS Martin MCO 50 plus (Tuttlingen, Germany) was used, with a power of 8 W.

Data collection

A telephone survey was conducted before treatment and at three and six months post-treatment, using a questionnaire that assessed vulvovaginal symptoms and their impact on daily life and sexuality and included additional information on sociodemographic aspects, satisfaction index indicators, and types of adverse effects from the treatment. Survey data were entered into Google Forms, creating a data matrix in MS Excel (Microsoft Corporation, Redmond, Washington), which was subsequently analyzed using IBM SPSS Statistics for Windows, Version 24 (Released 2016; IBM Corp., Armonk, New York).

Statistical analysis

Data were analyzed using SPSS software, version 24. Descriptive statistics were applied to summarize quantitative variables using means and standard deviations, while frequencies and percentages were calculated for qualitative variables. A paired t-test was performed to assess changes in dependent quantitative variables over time. A significance level of p < 0.05 was set for all statistical analyses to determine statistically significant differences.

## Results

The study population consisted of 32 patients, of whom 25 met the inclusion criteria. The average age of the participants was 57.76 ± 9.49 years. Among them, 20 patients (80%) were of urban origin, while the remaining five patients (20%) resided in rural areas. Educational background was varied: 10 patients (40%) had completed university studies, seven patients (28%) had completed high school, and four patients (16%) had achieved a technical or primary level of education (Table 1).

**Table 1:** Sociodemographic characterization of patients with GSM treated with fractionated CO2 laser. GSM: genitourinary syndrome of menopause; fr: frequency; %: percent; CO_2_: carbon dioxide

Sociodemographic Characteristics n=25	Fr	%
Marital status		
Single	10	40
Married	12	48
Stable de facto union	2	8
Widow	1	4
Origin		
Urban	20	80
Rural	5	20
Schooling		
Primary	4	16
High school	7	28
Technician	4	16
University	10	40

In terms of menopausal status, 22 patients (88%) were postmenopausal, and 15 patients (60%) reported being sexually active. Prior to undergoing laser treatment, 30% of the patients had been on hormone replacement therapy for six months or longer; specifically, six patients (24%) had received local therapy, while one patient (4%) had undergone systemic therapy (Table 2).

**Table 2:** Gynecological characteristics of patients with GSM treated with fractionated CO2 laser. GSM: genitourinary syndrome of menopause; fr: frequency; %: percent; CO_2_: carbon dioxide

Gynecological Characteristics n=25	Fr	%
Previous local hormone therapy	6	24%
Previous systemic hormone therapy	1	4%
Active sex life	15	60%
Postmenopause	22	88%
Age of menopause	2	8%
38 years	1	4%
41 years	2	8%
42 years	1	4%
43 years	1	4%
45 years	3	12%
47 years	4	16%
48 years	2	8%
49 years	6	24%
50 years	2	8%
Premenopause	3	12%

Regarding the symptoms of GSM, patients reported their symptoms as mild or moderate, with no severe symptoms noted. These symptoms were evaluated before treatment and at three and six months post-treatment. The distribution of symptom severity was as follows: mild 13, 24, and 25 patients at baseline, three months, and six months post-treatment, respectively; moderate 12 patients (48%), one patient (4%), and 0 patients at the same time points (Table 3).

**Table 3:** Severity of symptoms in patients who received fractional CO2 laser therapy for GSM management. GSM: genitourinary syndrome of menopause; fr: frequency; %: percent; CO_2_: carbon dioxide

Severity of Symptoms n=25	Fr	%
Prior therapy		
Mild	13	52%
Moderate	12	48%
Severe	0	0%
3 months post-therapy		
Mild	24	96%
Moderate	1	4%
Severe	0	0%
6 months post-therapy		
Mild	25	100%
Moderate	0	0%
Severe	0	0%

**Figure 1:**
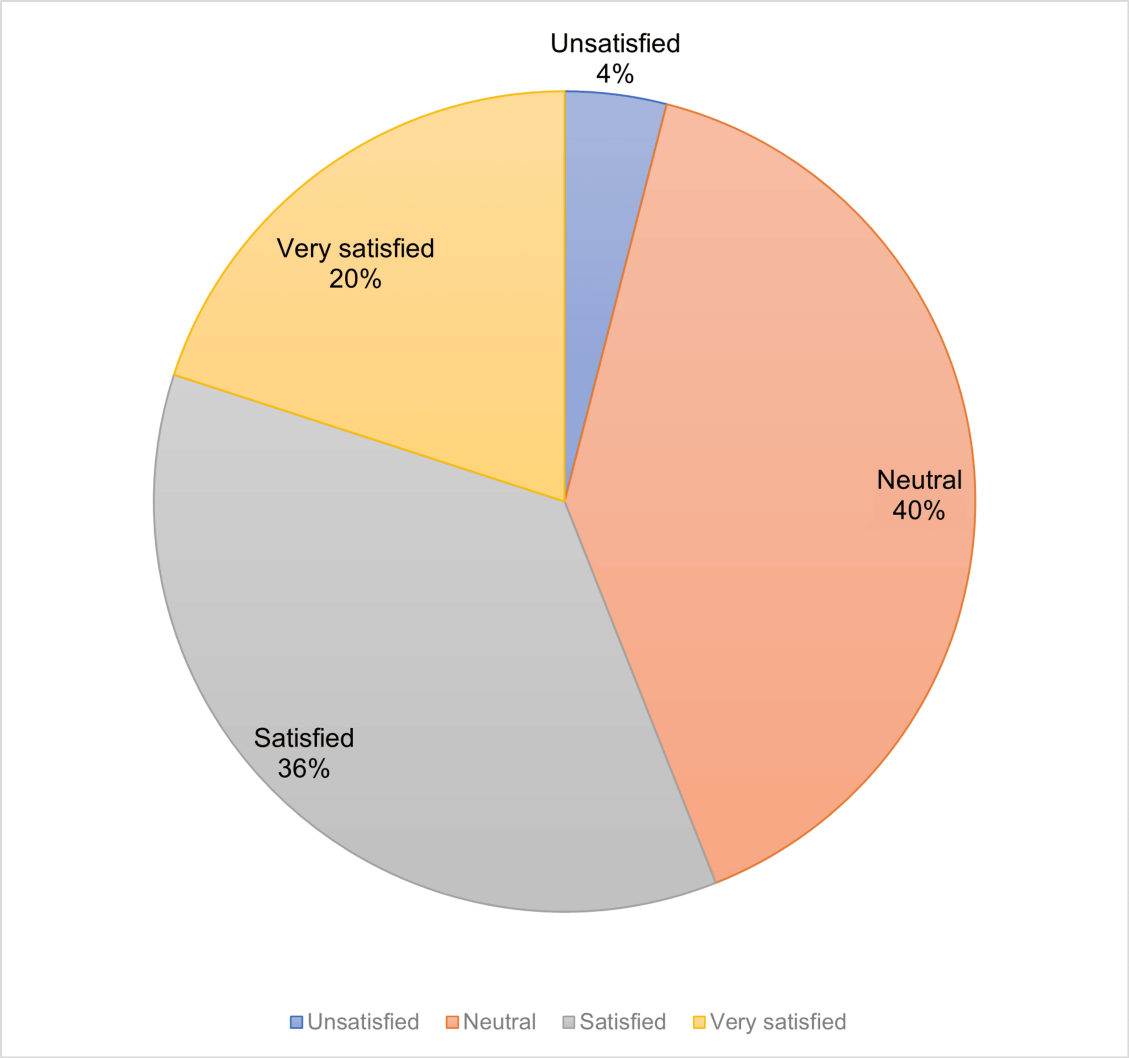
Satisfaction index (CSAT) of GSM patients treated with fractional CO2 laser. CSAT: customer satisfaction score; GSM: genitourinary syndrome of menopause Source: Data collection sheet

Satisfaction levels among patients were varied: 10 patients (40%) reported being neutral, nine patients (36%) were satisfied, five patients (20%) were very satisfied, and one patient (4%) expressed dissatisfaction (Table 4) (Figure 1). Additionally, 96% of the participants indicated they would recommend the therapy to other patients.

**Table 4:** CSAT of GSM patients treated with fractional CO2 laser. CSAT: customer satisfaction score; GSM: genitourinary syndrome of menopause; fr: frequency; %: percent; CO_2_: carbon dioxide

CSAT n=25	Fr	%
Unsatisfied	1	4%
Neutral	10	40%
Satisfied	9	36%
Very satisfied	5	20%

Adverse reactions to the administered therapy were reported by 60% of the patients. The adverse effects included pruritus in 10 patients (40%), pain in seven patients (28%), dyspareunia in six patients (24%), burning sensation in six patients (24%), and erythema in four patients (16%) (Table 5).

**Table 5:** Adverse effects reported by GSM patients treated with fractionated CO2 laser. GSM: genitourinary syndrome of menopause; fr: frequency; %: percent; CO_2_: carbon dioxide

Adverse Effects n=25	Fr	%
Adverse effects		
Yes	15	60%
No	10	40%
Pruritus	10	40%
Pain	7	28%
Dyspareunia	6	24%
Burns	6	24%
Erythema	4	16%
Swelling	0	0%

**Figure 2:**
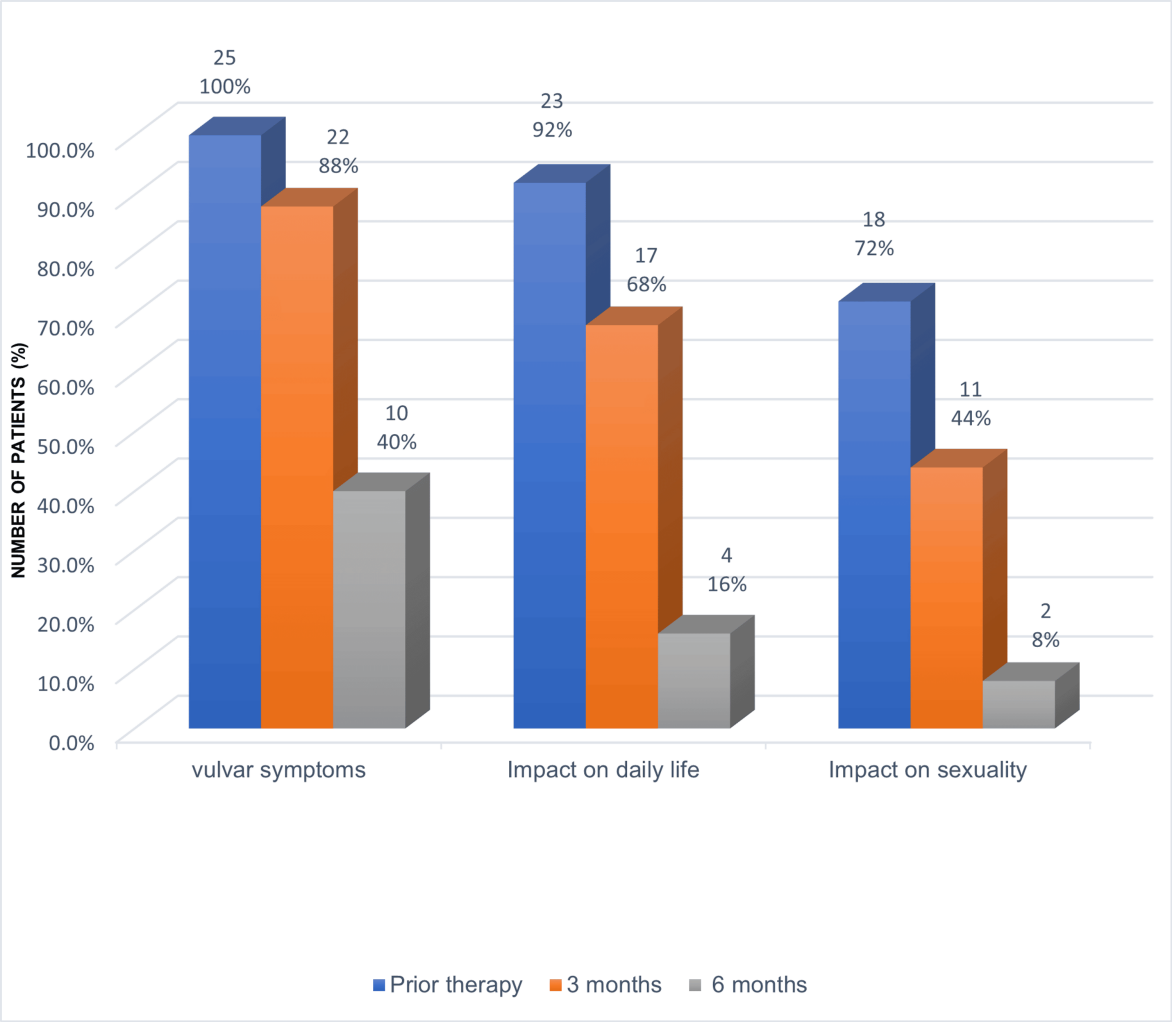
Evolution of GSM symptoms through the application of the VSQ at three and six months after laser CO2 treatment. GSM: genitourinary syndrome of menopause; Fr: frequency; % percentage; VSQ: Vulvovaginal Symptoms Questionnaire Source: Data collection sheet

Concerning the VSQ, each item was assessed before treatment and at three and six months post-treatment. The symptomatology and total scores are summarized in Tables 6-8 (Figure 2).

**Table 6:** Quality of life evaluation in GSM women treated with fractional CO2 laser. A p-value < 0.05 was considered statistically significant. GSM: genitourinary syndrome of menopause; fr: frequency; %: percent; CO_2_: carbon dioxide; VSQ: Vulvovaginal Symptom Questionnaire; DX: average difference

Questionnaire Components n=25	Prior Assessment		3 months		6 months	
	Fr	%	Fr	%	Fr	%
Vulvar symptoms	25	100	22	88	10	40
Before therapy compared to 3 and 6 months after treatment						
DX			4.7		6.7	
P-value			0.11		0.010	
Item 1. Vulvar vaginal pruritus	11	44	6	24	2	8
Item 2. Vulvar or vaginal burning	21	84	9	36	3	12
Item 3. Vulvar or vaginal pain	13	52	2	8	1	4
Item 4. Vulvar or vaginal irritation	12	48	2	8	0	0
Item 5. Vulvar or vaginal dryness	23	92	14	56	6	24
Item 6. Vulvar or vaginal secretions	0	0	0	0	0	0
Item 7. Vulvar or vaginal smell	0	0	0	0	0	0
Item 8. Concern about symptoms	6	24	1	4	0	0
Item 9. Appearance concern	0	0	1	4	0	0
Item 10. Frustration with vulvar symptoms	1	4	0	0	0	0
Item 11. Embarrassment from vulvar symptoms	0	0	0	0	0	0
Impact on daily life	23	92	17	68	4	16
Before therapy compared to 3 and 6 months after treatment						
DX			7		10.5	
P-value			0.122		0.063	
Item 12. Vulvar symptoms affect interaction with other people	17	68	2	8	2	8
Item 13. Affects the desire to be with other people	21	84	10	40	4	16
Item 14. Make it difficult to show affection	12	48	7	28	0	0
Item 15. Make it difficult to do or enjoy work	0	0	1	4	0	0
Impact on sexuality	18	72	11	44	2	8
Before therapy compared to 3 and 6 months after treatment						
DX			6.8		9.2	
P-value			0.010		0.017	
Item 16. Affects the desire for intimacy	10	40	1	4	0	0
Item 17. Affects sexual intercourse	15	60	6	24	0	0
Item 18. Cause pain during sexual activity	12	48	4	16	0	0
Item 19. Cause dryness during sexual activity	10	40	3	12	2	8
Item 20. Result in bleeding during sexual activity	1	4	0	0	0	0

**Table 7:** VSQ score in GSM patients treated with fractional CO2 laser. A p-value < 0.05 was considered statistically significant. GSM: genitourinary syndrome of menopause; fr: frequency; %: percent; CO_2_: carbon dioxide; VSQ: Vulvovaginal Symptom Questionnaire; DX: average difference

VSQ Components Score n=25	Prior Assessment		3 months		6 months	
	Fr	%	Fr	%	Fr	%
Vulvar symptoms (0–11 points)	X:4.1667		X:5 DX: -0.8		X:1.6667 DX: 2.5	
0 points			3	12%	15	60%
1 points	1	4%	13	52%	8	32%
2 points	2	8%	6	24%	2	8%
3 points	11	44%	2	8%	-	-
4 points	7	28%	1	4%	-	-
5 points	3	12%	-	-	-	-
6 points	1	4%	-	-	-	-
P-value	-		P=0.842		P=0.368	
Impact on daily life (0–4 points)	X:7.6667		X:5.6667 DX:2		X:1.3333 DX:6.3333	
0 points	2	8%	8	32,0%	21	84%
1 point	3	12%	14	56,0%	2	8%
2 points	13	52%	3	12,0%	2	8%
3 points	7	28%	-	-	-	-
P-value	-		P=0.789		P=0.161	
Impact on sexuality (0–5 points)	X: 4.5000		X:2.7500 DX:1.75		X:0.5 DX:4.0	
0 points	7	28%	14	56%	23	92%
1 points	3	12%	10	40%	2	8%
2 points	4	16%	-	-	-	-
3 points	7	28%	-	-	-	-
4 points	4	16%	1	4%	-	-
P-value	-		P=0.605		P=0.047	

**Table 8:** Evolution of GSM symptoms through the application of the VSQ at three and six months after laser CO2 treatment. A p-value < 0.05 was considered statistically significant. GSM: genitourinary syndrome of menopause; fr: frequency; %: percent; CO_2_: carbon dioxide; VSQ: Vulvovaginal Symptom Questionnaire

VSQ Component n=25	Prior Assessment		3 months		6 months	
	Fr	%	Fr	%	Fr	%
Vulvar symptoms	25	100	22	88	10	40
Impact on daily life	23	92	17	68	4	16
Impact on sexuality	18	72	11	44	2	8
0 and 3 months comparison						
Average difference	5.3					
P-value	0.047					
0 and 6-month comparison						
Average difference	16.6					
P-value	0.005					

Vulvar symptoms

**Figure 3:**
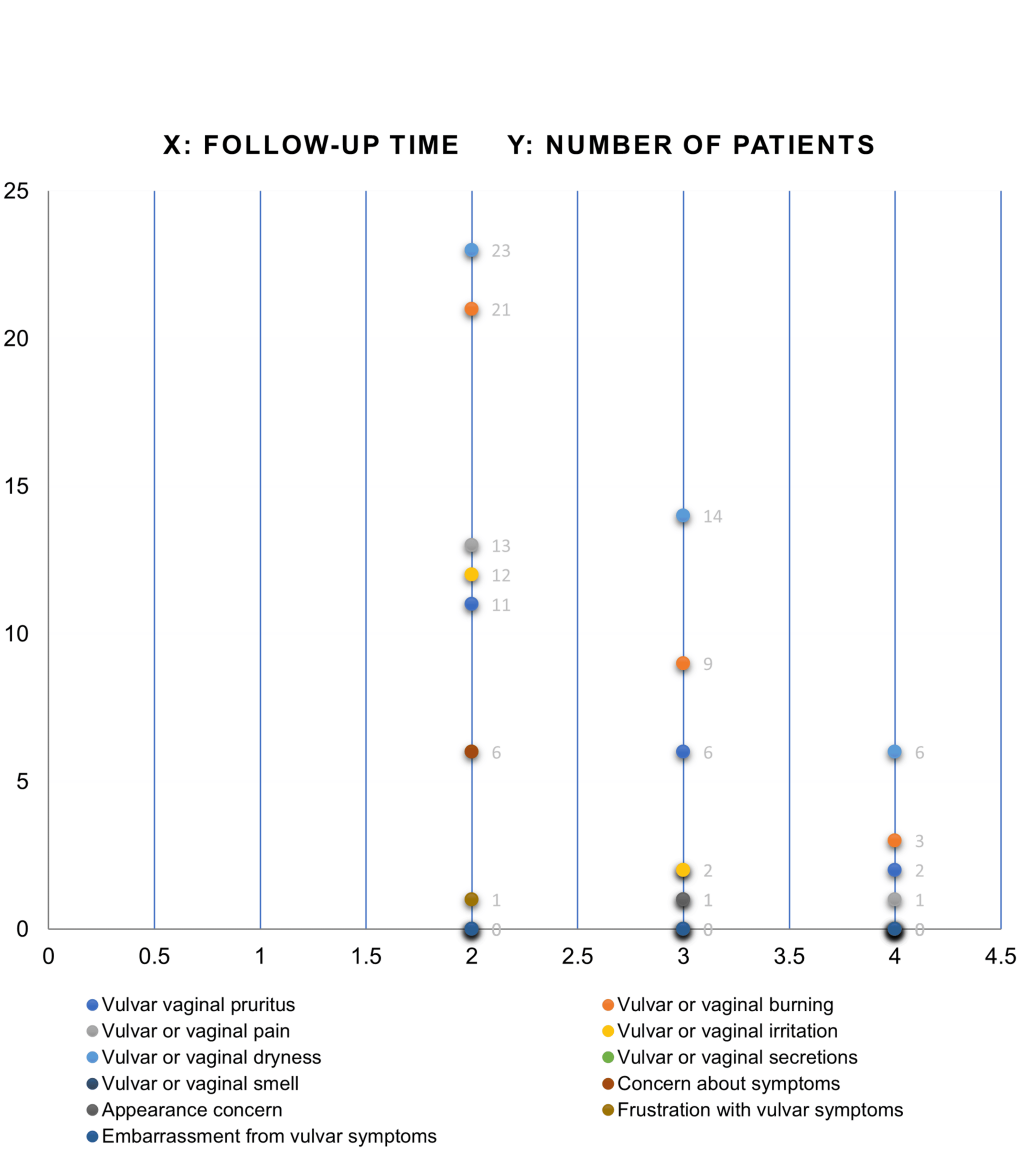
Vulvar symptoms evolution showing a downward trend after laser therapy. Three- and six-month follow-up.

Initially, 25 patients (100%) reported at least one vulvar symptom, which decreased to 22 patients (88%) at three months and to 10 patients (40%) at six months. The maximum vulvar symptom score prior to therapy was 6 out of 11, which reduced to 2 at six months, resulting in 15 patients (60%) being asymptomatic (Figure 3). The average score difference was 1.67, with a p-value of 0.368.

Impact on daily life

**Figure 4:**
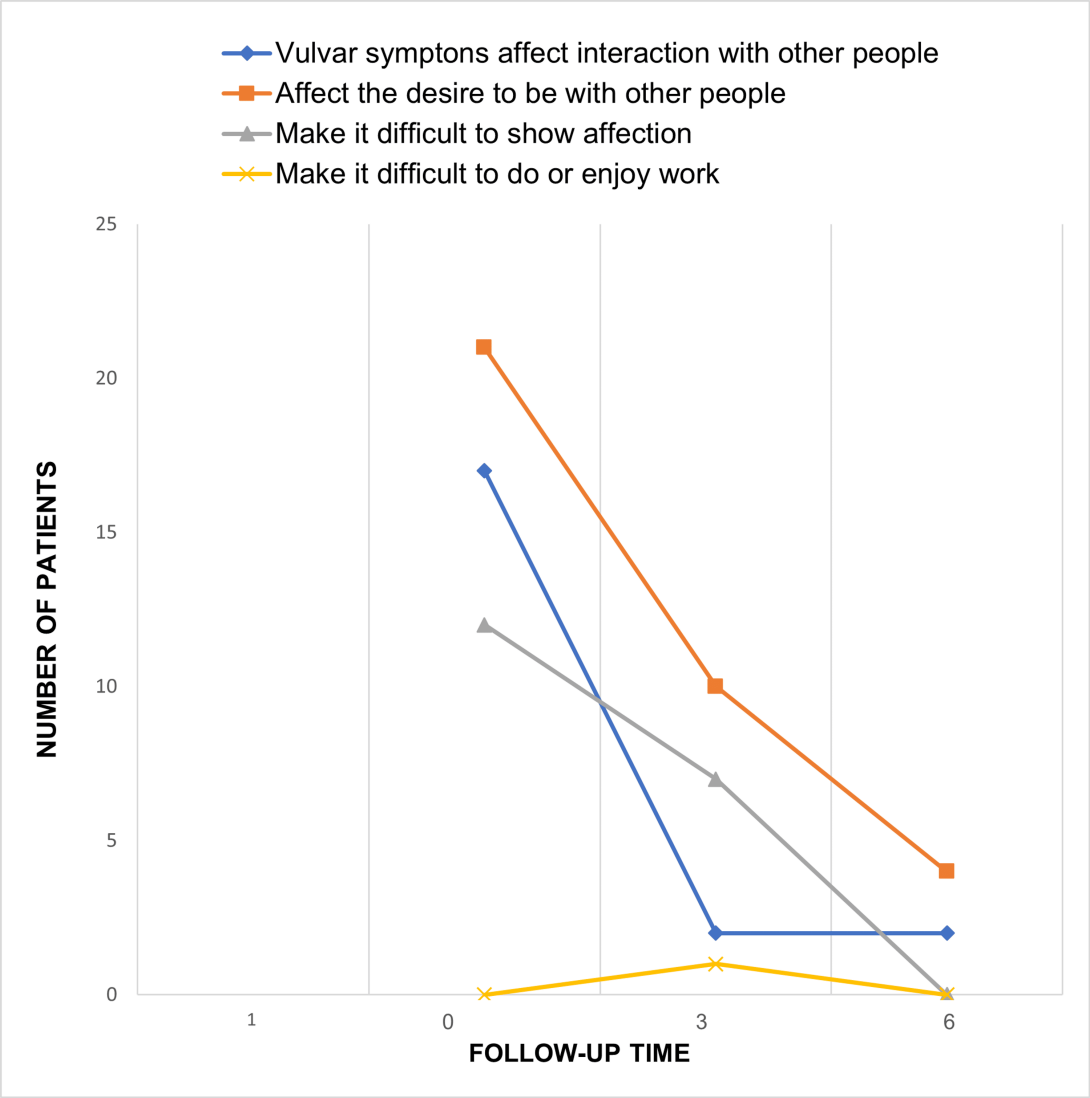
Impact of GSM on patients' quality of life showing a downward trend after laser therapy. Three- and six-month follow-up.

Prior to laser therapy, 23 patients (92%) reported that GSM negatively affected their daily lives. This figure decreased to 17 patients (68%) at three months and to four patients (16%) at six months. The maximum impact score prior to treatment was 3, reported by seven patients (28%), while at six months, only two patients (8%) reported a score of 2. The majority, 21 patients (84%), denied any impact on their quality of life at six months, although the average difference was not statistically significant (Figure 4).

Impact on sexuality

**Figure 5:**
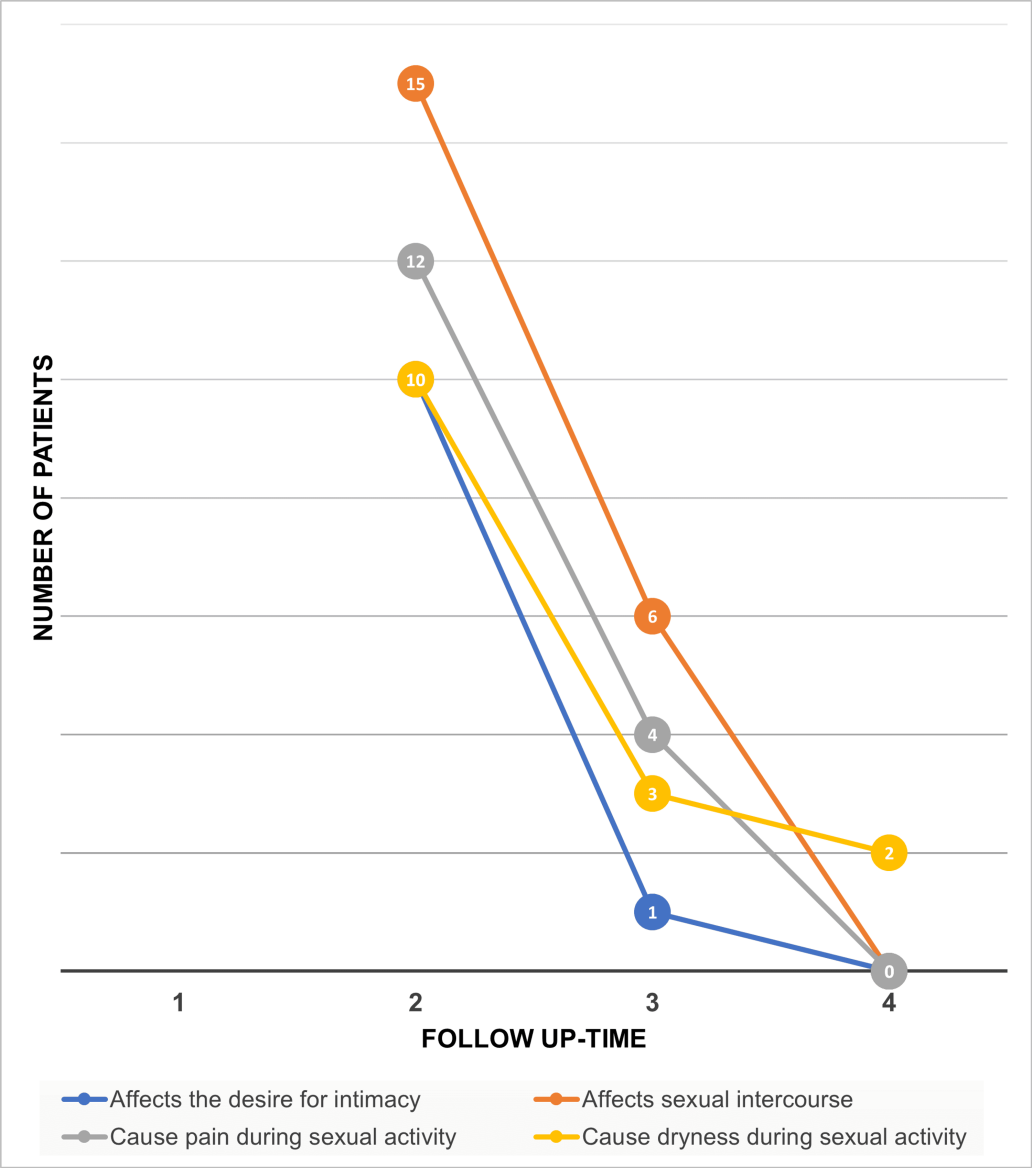
Impact on sexuality of the GSM demonstrating a downward trend after laser therapy. Three- and six-month follow-up.

Before treatment, 18 patients (72%) reported a negative impact on their sexual life, which decreased to 11 patients (44%) at three months and only two patients (8%) at six months (Figure 5). Regarding the scoring of this item (0 to 5 points), four patients (16%) had a score of 4, and seven patients (28%) had a score of 3 before therapy. At three months, 14 patients (56%) reported no impact, and by six months, 23 patients (92%) were free from this impact, with those affected scoring 1. The average difference between the initial assessment and the six-month follow-up was statistically significant, with a p-value of 0.047.

Table 9 presents the frequency of each item in the VSQ as reported by patients prior to therapy and at three and six months following laser treatment. A significant reduction in the number of patients experiencing vulvar symptoms and a diminished impact on quality of life were observed at six months. Additionally, reductions in the impact on sexuality were noted at both three and six months post-treatment. When evaluating the three components of GSM collectively, a significant decrease in the mean scores was observed at three months post-treatment, with an even greater reduction at six months.

**Table 9:** Total VSQ score in patients with GSM treated with fractional CO2 laser in the HMADB from June 2022 to October 2023. GSM: genitourinary syndrome of menopause; fr: frequency; %: percent; CO_2_: carbon dioxide; VSQ: Vulvovaginal Symptom Questionnaire; HMADB: Hospital Militar Escuela Dr. Alejandro Dávila Bolaños

Total Quiz Score (Points) n=25	Prior Assessment		Evaluation at 3 Months		Evaluation at 6 Months	
	Fr	%	Fr	%	Fr	%
0	-	-	1	4.0%	13	52.0%
1	1	4.0%	2	8.0%	9	36.0%
2	-	-	8	32.0%	-	-
3	-	-	10	40.0%	1	4.0%
4	1	4.0%	2	8.0%	2	8.0%
5	5	20.0%	1	4.0%	-	-
6	4	16.0%	-	-	-	-
7	2	8.0%	-	-	-	-
8	2	8.0%	1	4.0%	-	-
9	3	12.0%	-	-	-	
10	4	16.0%	-	-	-	-
11	2	8.0%	-	-	-	-
12	1	4.0%	-	-	-	-

## Discussion

As in the world population, GSM occurs in a large portion of patients treated in the gynecology service of "Dr. Alejandro Dávila Bolaños" Military Hospital, aged between 40 and 74 years, regardless of education or origin, affecting their quality of life. According to international literature, urogenital symptoms affect approximately 50% of women between the ages of 55 and 85. In 2016, the Chilean Journal of Gynecology and Obstetrics revealed that 50% of women over 60 years of age had symptoms related to GSM (dryness, pain, and dyspareunia, as well as affecting their sex life) [9].

The main objective of treatment in this pathology is to relieve the symptoms. For this purpose, vaginal lubricants, long-acting vaginal moisturizers, and systemic (oral and transdermal) and local hormone therapies (vaginal estrogen in low doses) can be used. The lack of response to these treatments and the contraindication or fear of the use of hormones, as observed in 30% of the sample, influence the search for alternative therapies. One of these therapies, and the one used for this study, is the fractional CO_2_ laser, described as a simple emerging therapeutic option that was well tolerated and without adverse effects [10].

The mechanism of action proposed is that this therapy promotes the regeneration of the vaginal epithelium by increasing its thickness and glycogen content. This process is driven by the activation of fibroblasts, which stimulate the production of new collagen and other extracellular matrix components within the lamina propria. These structural modifications enhance the elasticity and integrity of vaginal tissue, counteracting the effects of estrogen deficiency commonly seen in GSM. Furthermore, the treatment induces neovascularization, improving blood flow to the affected area and supporting tissue repair and hydration. These histological improvements align with significant clinical benefits, including reduced vaginal dryness, pain during intercourse, and other discomforts associated with GSM [11,12].

Similar to the diagnosis, the follow-up evaluation of GSM is clinical and is established primarily by a complete medical history and pelvic physical examination. Laboratory tests, cultures, or biopsies may be indicated if the vulvovaginal appearance is not typical or does not improve after therapy. Two genitourinary outcome measurement instruments are the VSQ and the Vaginal Aging Daily Impact Questionnaire.

The VSQ is a tool for detecting symptoms and the degree of emotional and sexual affectation that GSM entails [13] in such a way that it can be used for follow-up after therapy, including laser. In this study, it was applied before therapy and three and six months after the last laser session, allowing the impact on the patient's quality of life to be measured.

According to the score assigned to the VSQ, vulvar symptoms were present in 100% of the patients, with vulvar and vaginal dryness being the predominant symptoms (92%). This finding aligns with the report by Moral et al., where vaginal dryness was established as the most prevalent and annoying symptom, affecting 93% of women [14]. The impact on daily life was reported in 92% and on sex life in 72%. Six months after the last treatment session, the percentages of the VSQ were significantly reduced to 40%, 16%, and 8%, respectively, which is considered satisfactory and corresponds to what was reported in previous studies, including an uncontrolled trial of 30 women treated with three sessions of fractional CO_2_ laser, which reported significant improvements in dryness, dyspareunia, pain, burning, pruritus, and dysuria at the 12 weeks follow-up [14]. Similarly, Filippini et al. in 2020 performed a meta-analysis of the effectiveness of CO_2_ laser in women with vulvovaginal atrophy or GSM. All studies showed a significant reduction in symptoms (dryness, dyspareunia, pruritus, burning, and dysuria) [7].

Jang et al. compared CO_2_ laser therapy with vaginal estrogen therapy in a systematic review and meta-analysis. The study included six randomized clinical trials with 270 women and found no significant differences between the two treatments in terms of overall symptom improvement, as measured by the Vaginal Analog Scale, VHI, and FSFI [15].

Another way to assess the response to treatment was the degree of satisfaction of the users. So far, the satisfaction rates reported are high (Perino et al., 91.7%; Maggiore et al., 77.5%) [17,18]. In this research, however, 56% were between satisfied and very satisfied; however, only 4% were dissatisfied. 96% of the women affirmed their desire to recommend the therapy to other patients, indicating that it is a viable therapy.

To date, no significant safety concerns or adverse reactions to fractional CO_2_ lasers have been reported. An efficacy and safety study in 102 patients reported three cases of urinary tract infection and vaginal discharge, suggesting that this may reflect increased sexual activity rather than a direct consequence of the laser. Two women had postmenopausal bleeding, both with normal endometrial biopsies [18]. In this study, adverse effects occurred in 60% of the treated patients, being mild and transient (three to seven days); the most frequent was the presence of pruritus, which is why fractional CO_2_ laser is considered a therapy with a good margin of safety in expert hands.

The limitations of this study include its descriptive nature, as it evaluates treatment response without comparison to placebo or alternative therapies, thereby limiting conclusions on relative efficacy. Additionally, patient follow-ups were conducted for less than one year, which may not capture long-term outcomes or delayed adverse effects. Lastly, the small sample size may affect the generalizability of the findings and reduce the statistical power to detect smaller effect sizes.

## Conclusions

This study demonstrates that fractional CO_2_ laser therapy is a safe and effective non-hormonal treatment option that can significantly improve the quality of life for patients with GSM. Over six months, patients experienced meaningful relief from vulvar symptoms and reductions in the impact of GSM on daily life and sexual well-being, highlighting the potential of CO_2_ laser therapy for addressing this condition. Additionally, the VSQ proved an essential tool for tracking symptoms and quality of life, providing a clear and consistent way to monitor patient progress. The VSQ allowed for a more tailored approach to managing GSM, underscoring its value in future clinical practice.

As a pioneering step in exploring non-hormonal options for GSM, this study lays a foundation for further research across larger patient populations. With additional studies, the VSQ could become a standard tool in this area, supporting the establishment of a clinical protocol that incorporates fractional CO_2_ laser as a key treatment option. Such a protocol would equip clinicians with an evidence-based framework to better monitor and adjust treatments, ultimately improving patient outcomes. By advancing this non-hormonal approach, the findings here contribute to an evolving field focused on providing sustainable, personalized care options that address the varied health needs of menopausal patients. This study not only underscores the clinical promise of fractional CO_2_ laser therapy but also opens doors to a more comprehensive, patient-centered approach to managing GSM.
